# Unilateral Straight Hair—A Symptom of Acquired Horner's Syndrome in a Neonate

**DOI:** 10.1055/s-0038-1639479

**Published:** 2018-04-06

**Authors:** Chantal Ott, Andrei Bobylev, Stefan Gerhard Holland-Cunz, Johannes Mayr

**Affiliations:** 1 Department of Pediatric Surgery, University Children's Hospital Basel, Basel, Switzerland

**Keywords:** curly hair, Horner's syndrome, complication, lymphangioma, operation

## Abstract

A multicystic tumor of the right neck was detected in a girl at 29 weeks of gestation by fetal ultrasound and magnetic resonance imaging (MRI). The baby was delivered by cesarean section at week 37 of gestation. The newborn adapted well, with minimal compromise of breathing and drinking. Postnatal ultrasound and MRI revealed a cervical lymphangioma measuring 60.5 × 60.6 × 41.2 mm. We performed subtotal resection of the tumor when the girl was 34 days. As a complication of surgical resection, the girl developed ipsilateral Horner's syndrome. In the postoperative period, her curled hair turned straight at the side of the head affected by Horner's syndrome. At the age of 2.5 years, ultrasonic imaging revealed the presence of three cysts measuring 3 mm in diameter each. Horner's syndrome had improved, and the texture of the girl's hair had become curly again on both sides.

## Introduction

Pediatric Horner's syndrome is rare, occurring in 1.42 per 100,000 children below the age of 19 years. 1 It is a combination of symptoms that occur when the oculosympathetic pathway is interrupted. Horner's syndrome causes the clinical triad of miosis, ptosis, and anhidrosis on the ipsilateral side of the lesion.

The location of the lesion can be central (first neuron lesion), preganglionic (second neuron), or postganglionic (third neuron). 2 In children, mainly the preganglionic nerves are affected. 3

The etiology of pediatric Horner's syndrome can be divided into acquired and congenital causes. Acquired causes comprise surgery of the head, neck, and chest (42%). Horner's syndrome can also be caused by trauma, vascular malformations, neoplasms (e.g., neuroblastoma), and infections. About 15% of cases suffer from acquired causes other than surgery. 4

Congenital causes comprise birth trauma, neoplasms, infections, and carotid malformations, but often the cause of the lesion remains undefined. 5 In congenital Horner's syndrome or Horner's syndrome which occurred very early in life, heterochromia, which is a lighter colored iris in the affected eye, as well as Harlequin's sign, an asymmetric flush with hidrosis and redness of the face on the nonaffected side of the head, occur. 6

If Horner's syndrome is suspected, a cocaine test will confirm the diagnosis. To locate the lesion, a Paredrine test containing 4-hydroxyamphetamine, which normally results in noradrenaline release, is used. If the lesion is postganglionic, there is no dilatation because the transmitter vesicles cannot be released. In central or preganglionic lesions, mydriasis occurs because the postganglionic nerves remain intact and are able to produce noradrenaline. Because cocaine also blocks uptake of hydroxyamphetamine, these two tests should not be done on the same day.

Alternative methods to detect the location of the lesion comprise sweat test (searching for anhidrosis) and magnetic resonance imaging (MRI) scan. In congenital Horner's syndrome, pharmacological testing is difficult and often remains inconclusive. Thus, a contrast-enhanced MRI scan of the head, neck, and chest and an urinary catecholamine metabolite test to exclude the presence of neuroblastoma are often the only diagnostic tests applied. 6 7

Change of hair texture caused by Horner's syndrome is a rare phenomenon and has only been described sporadically in the literature. 8 All human hair fibers exhibit the same basic structure. However, it is the three-dimensional structure of the entire fiber that determines whether the hair appears straight or curled. According to recent research, hair shape is determined by genetic and biological factors. 9 A curled hair has an elliptical or “D”-like cross-section. During hair growth, the ellipse changes to form a coil.

Since the follicle of a highly curled hair appears curved, it is suggested that a curly follicle produces a curled hair, and asymmetry of the follicle results in curled hair formation regardless of ethnicity. However, it remains unknown what causes curved follicles and which factors are responsible for asymmetry of the hair follicle. 9 10

We report a rare case of ipsilateral straight hair in association with acquired Horner's syndrome in an infant and discuss the possible mechanisms for this phenomenon.

## Case Report

A Caucasian baby girl, weighing 3225 g, was delivered in the 37th week of gestation by cesarean section after amniotic fluid was lost. Ultrasonic examination during pregnancy showed a huge cervical mass. The diagnosis of a giant lymphangioma of the neck was suspected after MRI.

**Fig. 1:**
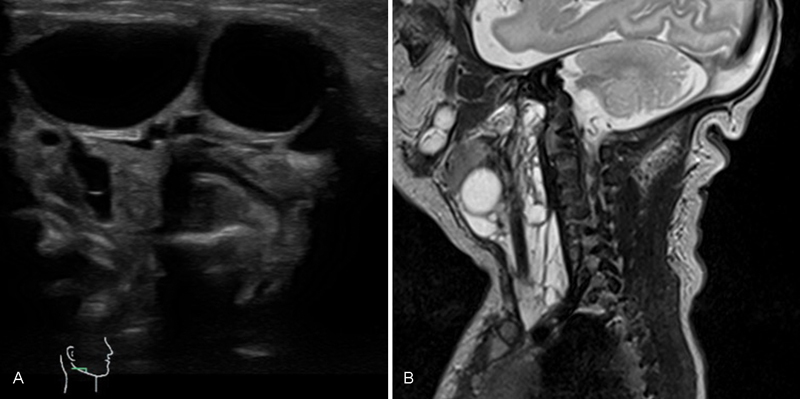
( **A** ) Duplex sonography of the right neck region obtained on the first day of life showing the vertebral artery and multiple hypoechogenic cysts of different size representing a mixed-type lymphangioma. ( **B** ) Magnetic resonance imaging scan of the neck region showing multiple cysts of different size extending from the skull base to the upper thoracic aperture in the anterior region of the neck.

Ultrasonic and MRI scans showed the mass to be composed of multiple cysts of different size with a few solid parts ( Fig. 1A , B ). It was located predominantly on the right side of the infant's neck, extending over the midline anteriorly. The tumor caused a tilting of her neck to the left. Because there was no massive compression of the trachea, we decided to postpone surgery beyond the neonatal period. The girl was discharged from hospital at the age of 6 days with home pulsoxymetric surveillance. Regular visits to the outpatient clinic were arranged.

### Operative Intervention

During the operation at week 5 of the infant's life, we noted that the main vessels and nerves on the right side of the neck were completely entrapped by the multicystic lymphangioma. We undertook a subtotal excision of the mixed-type lymphangioma, due to the presence of dense adhesions between the lymphangioma and vessels and nerves at the right side of the neck.

Small remnants of the cystic lymphangioma in the area of the upper thoracic aperture and in close vicinity to the trachea, skull base, and recurrent laryngeal nerve were not removed. We preserved the trunk of the transverse cervical nerve, but severed some minor peripheral nerve branches to facilitate the removal of the central part of the lymphangioma.

Eight days after surgery, the girl was able to breath and drink without any difficulties and was discharged from hospital. The surgical wound healed well. The patient's parents reported that her voice was moderately weakened. We opted against a laryngoscopic examination because the weakening of the voice improved steadily.

**Fig. 2:**
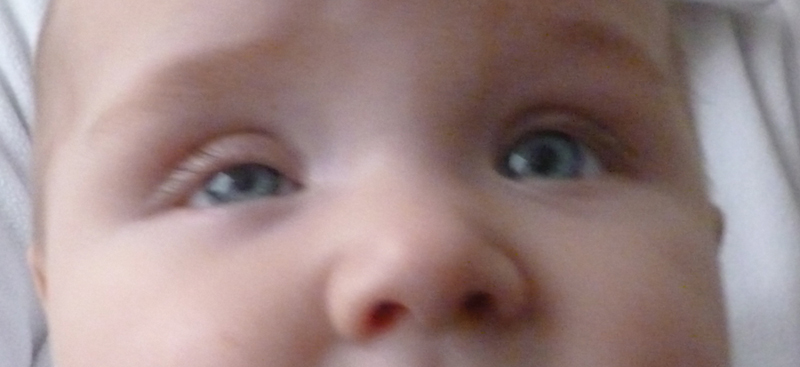
Photograph taken 4 months after subtotal resection of the lymphangioma. Miosis and ptosis of the eye affected by Horner's syndrome. There is no heterochromia of the iris.

Postoperatively, we noted that the girl's right eyelid was positioned lower than her left eyelid, and her right palpebral fissure was smaller when compared with the left ( Fig. 2 ). We diagnosed Horner's syndrome and referred her to the ophthalmologists for diagnostic workup and treatment. Horner's syndrome was confirmed. The infant showed partial ptosis of her right eyelid, and the diameter of the right pupil was smaller when compared with the left eye, with preserved pupillary light reflex. In addition, we prescribed physiotherapy to improve the moderate limitation of active elevation and abduction of her right shoulder and arm and to correct the tilting of her neck.

At the age of 3 months, the residual swelling of the infant's neck on the right side had subsided spontaneously, and her voice had recovered. However, Horner's syndrome was still present but showed further improvement. Movement of her right arm and shoulder had improved.

**Fig. 3:**
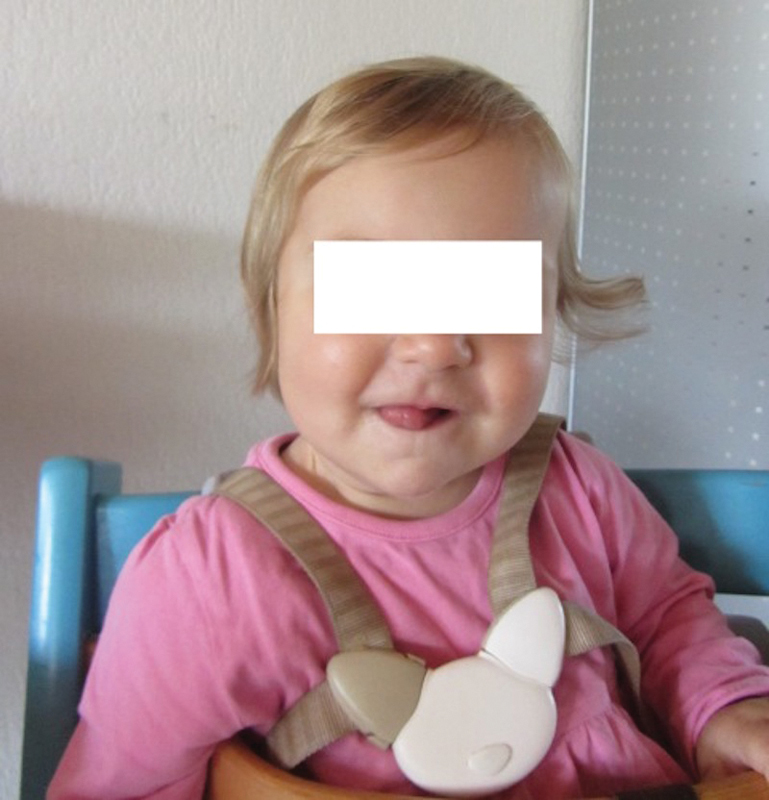
Photograph taken 6 months after the operation. Straight hair is visible on the right side of the head. The right side of the head is affected by Horner's syndrome. The original hair texture of the infant is retained on the left side of the head.

**Fig. 4:**
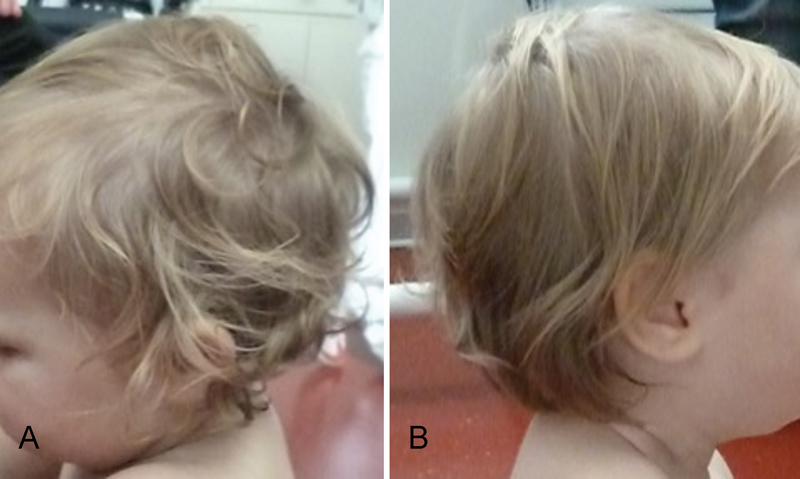
Photograph taken at 16 months. ( **A** ) Straight hair on the right side of her head which is affected by Horner's syndrome. ( **B** ) The original hair texture of the infant is retained on the left side of the head which is not affected by Horner's syndrome.

When the girl was 6 months old, her parents reported that her hair texture had changed. At birth, her hair was brown and straight. After birth, the color of the hair turned from brownish to blond, and the hair texture became more curled on both sides of the head. Four months after surgery, the hair on the left side of her head was still curly, while the hair on the right side had turned straight ( Figs. 3 , 4A , B ).

At the age of 9.5 months, the ophthalmologist reported that ptosis had improved and the girl's vision had remained stable with no noticeable difference in the visual acuity between her eyes.

We noted a minimal facial asymmetry due to her lymphangioma on the right side. Movement of her right shoulder and arm showed minimal restriction of elevation and abduction. Her psychomotor development was normal for age.

Follow-up MRI scan of the neck at the age of 1 year revealed a small residual part of the lymphangioma in close vicinity of the larynx, and we therefore opted against sclerotherapy. The residual cysts became smaller spontaneously.

At the age of 2.5 years, ultrasonic imaging showed several cysts measuring 3 mm that were localized close to her right thyroid lobe. Horner's syndrome had improved further but moderate ptosis of the right eyelid was still present. There was no impairment of her visual acuity. Her hair had normalized, and the hair texture was curly on both sides of her head. The tilting of the neck had subsided, and we noted no scoliosis. At this point, we stopped physiotherapy.

## Discussion

Our patient showed some complications after surgery in the neck region. The transient weakening of the voice had most likely occurred due to the preparation of the laryngeal recurrent nerve on the affected side. 11 The laryngeal recurrent nerve was running in close vicinity of the medial circumference of the lymphangioma. The weakening of the voice improved steadily with time.

The motor impairment of the ipsilateral shoulder was caused by partial weakness of the upper part of the trapezius muscle. This part is innervated mainly by the spinal accessory nerve and branches of the cervical plexus. 12 Muscle function improved with physiotherapy over time. We, therefore, did not conduct neurophysiologic examination.

The infant developed unilateral Horner's syndrome after subtotal excision of a large cervical lymphangioma of the neck. In addition to the usual symptoms, our patient experienced a striking symptom: her curly hair turned nearly straight on the side of the head affected by Horner's syndrome. We hypothesized that this phenomenon was associated with Horner's syndrome and was not due to spontaneous change of hair texture. The change in hair structure was not obvious until a few months after surgery. Other authors did not mention at what time point the change of hair structure appeared. 2 3 8 We do not believe that this phenomenon was due to spontaneous change of hair texture during growth of the child because this would take longer than just a few months. 10

We are not aware of the exact pathophysiological mechanism that caused the unilateral change of hair curliness in our patient. Shewmon 13 speculated that the reason for ipsilateral straight hair might be the loss of trophic effect of sympathetic stimulation. Similarly, the loss of trophic effect on the melanocytes in the iris results in a paler color of the iris.

Another investigation, partly based on a study in sheep, suggested that the arrectores pilorum muscles may cause curly hair by rhythmic contractions. These muscles are innervated by sympathetic neurons. Thus, it seems reasonable that sympathetic denervation is associated with the occurrence of straight hair. 14 We consider this hypothesis somewhat unlikely since Thibaut et al 15 demonstrated, in 2007, that the arrectores pilorum muscles are not involved in the formation of curly hair. As part of the study, they separated the hair follicles from the muscles and sebaceous glands and implanted them in vitro, but the hair still grew curly. These authors concluded that the human hair shape is probably programmed by the hair bulb located at the basal area of the hair follicle. 15 Thus, the way how sympathetic nerves influence hair structure at the level of the hair follicles remains undefined. Similarly, it is not known at what age a lesion in the sympathetic pathway can lead to a change in hair curliness.

Reports in the medical literature exclusively describe children aged between 3 weeks and 2 years suffering from this hair texture phenomenon, and in contrast to our patient, all children suffered from congenital Horner's syndrome. Congenital Horner's syndrome is defined as a disorder manifesting in the first month after birth. 8 However, we cannot exclude the possibility that Horner's syndrome was caused by the lymphangioma in our patient. Occurrence of Horner's syndrome due to neuroblastoma has been described in the literature. 16 Our patient showed no sign of Horner's syndrome before surgery nor was her iris involved which represents a typical symptom of congenital Horner's syndrome. 16

Previously described children with change of hair texture exhibited similar features, with the exception of a 10-month old girl. The girl suffered from right-sided Horner's syndrome and was later diagnosed with neuroblastoma. 17 After thoracoscopic resection and chemotherapy, the patient developed alopecia. Six months after treatment start, her hair had partially regrown, but only on the left side of her head. To our knowledge, this is the first report of unilateral hair growth in a patient suffering from Horner's syndrome. 17

We found one literature report on a mild phenotype of giant axonal neuropathy which resulted in a change of hair texture in affected children. 18 This phenomenon in giant axonal neuropathy may be linked to a change of hair texture in acquired Horner's syndrome in young children.

To the best of our knowledge, there are no reports on adults who experienced change in hair texture due to acquired Horner's syndrome. Therefore, we assume that the loss of sympathetic stimulation has no significant influence on hair texture in adults. However, change of hair texture from curly to straight hair has been described after the treatment of hepatitis C virus infection and alopecia. 19 20 Inhibition or mutation of the epidermal growth factor receptor has been reported to change the hair texture and causing eyelash trichomegaly 21 In adults, a change of hair texture occurs very rarely, and causes are unrelated to Horner's syndrome. We report the rare case of an infant in whom Horner's syndrome occurred after surgical resection of a giant lymphangioma of the neck region. Horner's syndrome was accompanied by an ipsilateral change in hair texture. The change of hair texture showed gradual regression, and also Horner's syndrome improved with time. The pathway by which Horner's syndrome influences hair texture remains poorly understood. Further investigations are required to examine the cause of this phenomenon.

## Conclusion

Pediatricians should be aware that unilateral straight hair in children may be a symptom of Horner's syndrome.
